# Upregulation of Mas-related G Protein coupled receptor X2 in asthmatic lung mast cells and its activation by the novel neuropeptide hemokinin-1

**DOI:** 10.1186/s12931-017-0698-3

**Published:** 2018-01-03

**Authors:** Wichayapha Manorak, Chizobam Idahosa, Kshitij Gupta, Saptarshi Roy, Reynold Panettieri, Hydar Ali

**Affiliations:** 1 Department of Pathology, School of Dental Medicine, University of Pennsylvania, 240 South 40th Street, Philadelphia, PA 19104-6030 USA; 2 Department of Oral Medicine, School of Dental Medicine, University of Pennsylvania, Philadelphia, PA USA; 3 Present Address: Departmentof Oral and Maxillofacial Pathology, Medicine and Surgery, Temple University Kornberg School of Dentistry, Philadelphia, PA USA; 4 Rutgers Institute for Translational Medicine and Science, Rutgers University, New Brunswick, NJ USA

**Keywords:** Hemokinin-1, Substance P, Lung mast cells, MRGPRX2, NK-1R, Asthma

## Abstract

Hemokinin-1 (HK-1) is a novel neuropeptide produced by human bronchial cells and macrophages and causes contraction of human bronchi ex vivo. It is also generated by antigen/IgE-activated murine mast cells (MCs) and contributes to experimental chronic allergic airway inflammation via the activation of the neurokinin receptor-1 (NK-1R) expressed on murine MCs. We found elevated MC numbers in the lungs of individuals who died from asthma (asthma) when compared to lungs of individuals who died from other causes (non-asthma). Mas-related G Protein coupled receptor X2 (MRGPRX2) is a novel G-protein coupled receptor (GPCR) that is expressed predominantly on human MCs. We detected low level of MRGPRX2 in non-asthma lung MCs but its expression was significantly upregulated in asthma lung MCs. HK-1 caused degranulation in a human MC line (LAD2) and RBL-2H3 cells stably expressing MRGPRX2 and this response was resistant to inhibition by an NK-1R antagonist. However, knockdown of MRGPRX2 in LAD2 cells resulted in substantial inhibition of HK-1-induced degranulation. These findings suggest that while HK-1 contributes to the development of experimental asthma in mice via NK-1R on murine MCs the effect of this neuropeptide on human bronchoconstriction likely reflects the activation of MRGPRX2 on lung MCs. Thus, development of selective MRGPRX2 antagonists could serve as novel target for the modulation of asthma.

## Introduction

Hemokinin-1 (HK-1) is a newly discovered neuropeptide, which is produced by human bronchial cells and lung macrophages and causes contraction of isolated human bronchi [1]. In vivo studies demonstrated that HK-1 activates NK-1R on murine MCs and functions as an adjuvant for IgE-mediated anaphylaxis and lung inflammation in a MC-dependent model of chronic asthma [2]. Surprisingly, NK-1R antagonists, which are highly effective in modulating experimental allergic inflammation and airway hyperresponsiveness in mice, lack efficacy in the clinic [3–6]. The reason for this discrepancy is unknown.

Human skin MCs and a human MC line, LAD2 cells express cell surface MRGPRX2 and respond to the neuropeptide substance (SP) for signaling and degranulation [7]. Although human lung-derived cultured MCs express MRGPRX2 mRNA, they do not express the recept or on the cell surface and are unresponsive to SP for degranulation [7]. The possibility, however, that MRGPRX2 protein is expressed on primary human lung MCs has not been determined. The purpose of this study was: (a) to utilize double immunofluorescence technique to compare MRGPRX2 expression on lung MCs of individuals who died from asthma with those who died from other causes and (b) to determine if HK-1 induces degranulation in human MCs via MRGPRX2.

## Methods

### Lung samples and Immunofluorescence analysis

Lung samples from individuals who died from complications of asthma and control subjects who died from other causes were obtained from either the National Disease Resource Interchange (NDRI) or International Institute for the Advancement of Medicine (IIAM) and its use was approved by Institutional Review Board at Rutgers University. All donor tissue samples were harvested anonymously and de-identified. Thus, the use of these samples does not constitute human subjects research.

Deparaffinized lung sections were blocked with 5% donkey serum and immunofluorescence staining of MCs and MRGPRX2 was performed as described [8]. Images were captured on a Nikon Eclipse microscope with an Olympus digital microscope camera using 20X and 40X objectives. The number of MCs and MRGPRX2-positive MCs were counted in the 3 visual fields and the average of the 3 fields was taken as the final count for each sample. The image size and dimensions were exactly the same for all analyzed images. For measurement of MRGPRX2 intensity equal area was selected in 100 non-asthma and asthma lung MCs and their intensity was measured by ImageJ software.

### Transfection, flow cytometry and MC degranulation

Generation of RBL-2H3 cells stably expressing MRGPRX2 was described previously [9]. To detect cell surface MRGPRX2 expression in transfected RBL and native LAD2 cells, PE conjugated anti-human MRGPRX2 antibody was used. Cells were washed, fixed and analyzed on BD LSR II flow cytometer. For transient expression of NK-1R, RBL-2H3 cells (1 × 10^6^) were transfected with plasmids encoding NK-1R plasmid (2 μg), using the Amaxa nucleofector kit. After 24 h, cells were incubated anti-NK-1R antibody for 30 min at 4°C followed by Goat anti-rabbit IgG-PE, fixed and analyzed on BD LSR II flow cytometer. Degranulation in transfected RBL-2H3 cells and LAD2 cells was determined as described previously [9, 10].

### Knockdown of MRGPRX2 in LAD2 cells

Lentivirus shRNA-mediated knockdown of MRGPRX2 in LAD2 cells was performed as described previously [10]. Cell lysates prepared from control and MRGPRX2 knockdown cells were separated in SDS-PAGE gel, proteins were transferred on to nitrocellulose membrane and incubated with anti-MRGPRX2 antibody (Novus Biologicals, dilution 1: 500). This was followed by incubation with anti-rabbit secondary antibody and developed by West Pico chemiluminescent substrate.

## Results

**Fig. 1:**
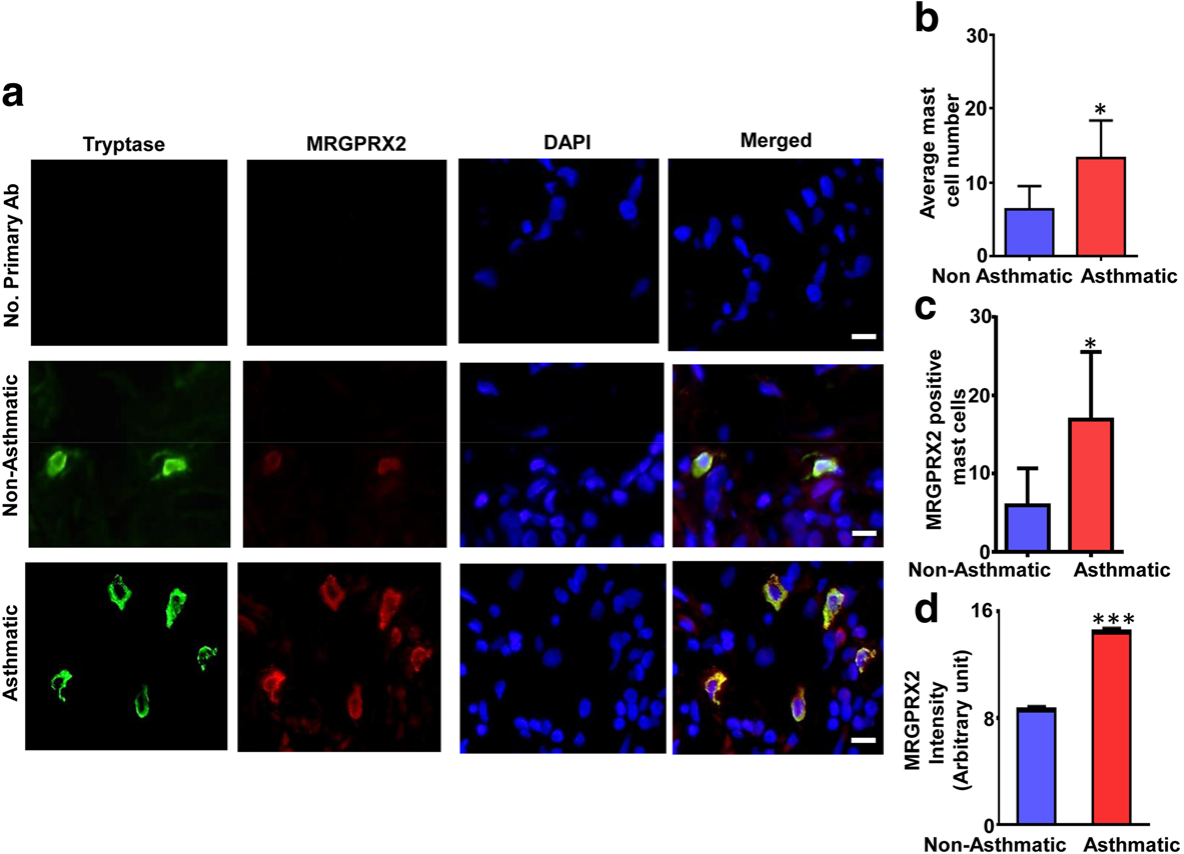
MRGPRX2 expression in human non-asthma and asthma lung. **a** Representative photomicrographs (*n* = 6) of immunofluorescence staining of human non-asthma and asthma lungs. Samples were stained with anti-human tryptase (green) and anti-MRGPRX2 antibody (red). Overlay of double stained samples are shown. Scale bar = 50 μm. **b** anti-tryptase stained slides were counted and data are presented as the number of MCs from 6 non-asthma and 6 asthma lungs. **c** MRGPRX2-positive MC number was counted in non-asthma vs asthma lung MCs and the data was represented as mean ± SEM of six lung samples. **d** MRGPRX2 intensity in 100 normal and asthma lung MCs was quantified using ImageJ software and data is represented as mean ± SEM. Statistical significance was determined by two-way ANOVA with Bonferroni’s post test

It is well documented that MC numbers are increased in the lungs of asthmatics when compared to lungs with other conditions [11]. Using anti-tryptase antibody, we confirmed that MC numbers are significantly elevated in asthma lung when compared to non-asthma lung (Fig. 1a, left panel green cells and Fig. 1b). We detected faint staining for MRGPRX2 in non-asthma lung, but intense staining was observed in asthma lung (Fig. 1a, red cells). Overlay of tryptase (green) and MRGPRX2 (red) showed all MCs in asthma lung express MRGPRX2 (Fig. 1a). We found that number of MRGPRX2-positive MCs are significantly greater in asthma lungs when compared with non-asthma lungs (Fig. 1b and c). Furthermore, the intensity of MRGPRX2 staining was significantly increased in asthma in lung MCs when compared to non-asthma lung MCs (Fig. 1d).

**Fig. 2:**
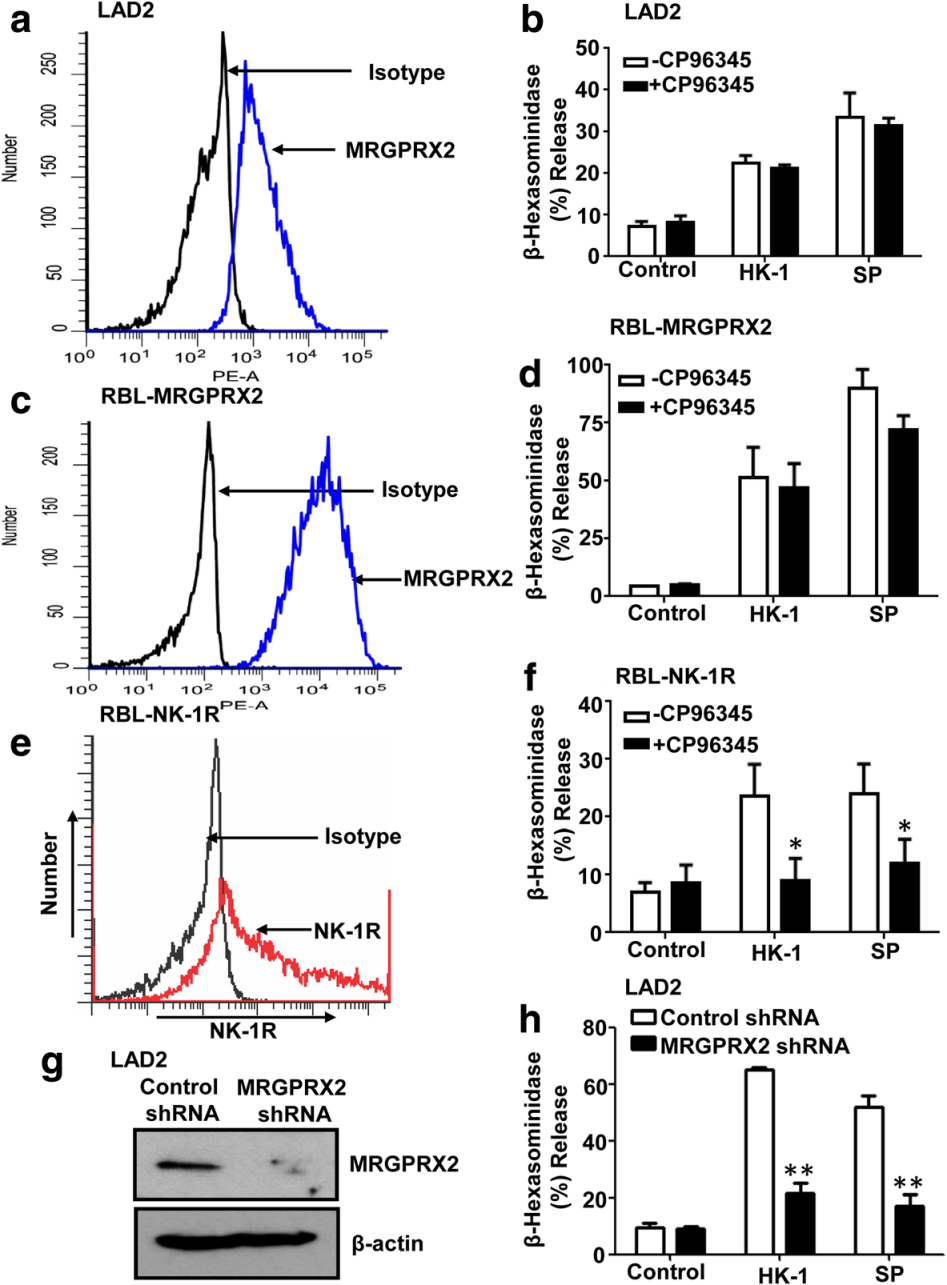
Role of MRGPRX2 on HK-1-induced degranulation in LAD2 cells and transfected RBL-2H3 cells. **a** Expression of MRGPRX2 on LAD2 cells **b** Effects of NK-1R antagonist CP96345 (10 μM) on SP (1 μM) and HK-1 (3 μM)-induced degranulation in LAD2 cells. **c** MRGPRX2 expression on RBL-2H3 cells stably expressing the receptor (RBL-MRGPRX2). **d** Effects of CP96345 (10 μM) on SP (1 μM) and HK-1(3 μM)-induced degranulation in RBL-MRGPRX2 cells. **e** Transient expression of NK-1R in RBL-2H3 cells (RBL-NK-1R) and **f** Effects of CP93645 on SP (1 μM) and HK-1 (3 μM) on degranulation in RBL-NK-1R cells. **g** Western blotting to determine the expression of MRGPRX2 in control shRNA and MRGPRX2 shRNA-transduced LAD2 cells. β–actin was used as a loading control. **h** Effects of shRNA-mediated knockdown of MRGPRX2 on SP (0.3 μM) and HK-1 (3 μM)-induced degranulation in LAD2 cells. Flow cytometry (**a**, **c** and **e**) and Western blotting data (**g**) presented are representative of 3 similar experiments. Degranulation data (**b**, **d**, **f** and **h**) are the mean ± SEM of 3 independent experiments. Statistical significance was determined by two-way ANOVA with Bonferroni’s post test. *p < 0.01

Although NK-1R is expressed on murine MCs it is not found on the surface of human skin and lung MCs [2, 7]. This suggests that if HK-1 induces degranulation in human MCs, it does so via the activation of a different GPCR. The neuropeptide substance P (SP) activates human culture-derived MCs, LAD2 cells and skin MCs via MRGPRX2 [7]. To determine if HK-1 activates human MCs via MRGPRX2 we used LAD2 cells, which endogenously express MRGPRX2 and RBL-2H3 cells stably expressing the receptor (RBL-MRGPRX2) [9, 10]. In both cases, we confirmed cell surface expression of MRGPRX2 by flow cytometry (Fig. 2a and c). Furthermore, SP and HK-1 caused degranulation in both cell types but this response was resistant to inhibition by the NK-1R antagonist CP96345 (Fig. 2b and d). Both SP and HK-1 induce Ca^2+^ mobilization in HEK293 cells transiently expressing NK-1R [12]. To validate the antagonist activity of CP96345 for NK-1R, we tested its effect on degranulation in response to HK-1 and SP in RBL-2H3 cells transiently expressing NK-1R (RBL-NK-1R) (Fig. 2e). As shown in (Fig. 2f), CP96345 caused substantial inhibition of HK-1 and SP-induced degranulation under condition at which it had no effect in LAD2 or RBL-MRGPRX2 cells (Fig. 2b and d). To further confirm the role of MRGPRX2 on HK-1-induced MC degranulation, we used lentivirus shRNA to silence receptor expression in LAD2 cells. Western blotting analysis confirmed knockdown of MRGPRX2 expression in LAD2 cells (Fig. 2g). Furthermore, degranulation in response to SP (0.3 μM) or HK-1 (3 μM) was significantly reduced (*p* ≤ 0.01) in MRGPRX2 knockdown cells when compared to control shRNA transduced cells (Fig. 2h).

## Discussion

Although studies with animal models have provided significant insights on the mechanisms of asthma, many of these models do not fully represent the disease process in human [13]. Not surprisingly, many therapeutic strategies developed using these models lack efficacy in human [13, 14]. In this regard, NK-1R signaling by HK-1 is necessary for FcεRI-MC-dependent experimental chronic airway inflammation in mice [2]. However, specific NK-1R antagonists that inhibit allergen-induced bronchoconstriction and airway inflammation in rodents lack efficacy in the clinic [3, 15]. These findings suggest that the failure of NK-1R antagonists in the clinic reflects the utilization of different GPCRs by SP and HK-1 in human versus murine MCs. In present study, we provide the first demonstration that expression of MRGPRX2 is upregulated in lung MCs in asthma when compared to non-asthma lung MCs and that both SP and HK-1 activate human MCs via MRGPRX2.

Previous reports indicated that human skin MCs express MRGPRX2 but lung MCs do not [7, 16]. It is noteworthy that only ~21% of human skin MCs obtained from control subjects express MRGPRX2. This increases to ~47% in skin MCs of patients with chronic urticaria without a change in total number of MCs [7]. In contrast, we found that almost all MCs in non-asthma lungs express MRGPRX2 at low level and this likely explains the resistance of these cells to SP for degranulation [7]. However, the number of MRGPRX2-positive MCs is significantly elevated in asthma lung when compared to non-asthma lung. In addition, the intensity of MRGPRX2 staining is significantly higher in asthma when compared to non-asthma lung MCs. These findings suggest that unlike the situation with skin MCs in chronic urticaria, both the number of MRGPRX2-expressing lung MCs and the level of receptor expression are upregulated in asthma.

Fujisawa et al., [7] showed that shRNA-mediated knockdown of MRGPRX2 resulted in substantial loss of SP-induced degranulation in human skin MCs but NK-1R antagonist (CP-96345) had no effect. The human MC line, LAD2 cell has been extensively used to the study of MRGPRX2 regulation [7, 8]. The data presented herein confirms the previous findings that SP activates human MCs via MRGPRX2 and suggests that HK-1 utilizes the same receptor for MC degranulation. This contention is supported by the following observations. First, as for SP [9], HK-1 induces robust degranulation in RBL-2H3 cells stably expressing MRGPRX2. Second, although the NK-1R antagonist CP93645 inhibited HK-1 and SP-induced degranulation in RBL cells expressing NK-1R, it had no effect on degranulation in LAD2 cells or RBL cells expressing MRGPRX2. Third, shRNA-mediated knockdown of MRGPRX2 resulted in substantial inhibition of both SP and HK-1-induced degranulation in LAD2 cells. Although NK-1R is the classic GPCR for neurokinins such as SP and HK-1 [12], it now appears that MRGPRX2 also acts as a neurokinin receptor [7]. It is important to note that while NK-1R is expressed in a variety of cell types including murine MCs, MRGPRX2 is predominantly expressed in human MCs [2, 7, 12]. This may explain why classic NK-1R antagonists, which effectively modulate experimental allergic and inflammatory responses in mice, lack efficacy in the clinic [3–6].

## Conclusion

This study provides the first demonstration that both the number of MRGPRX2-expressing MCs and the level of receptor expression are significantly upregulated in lung MCs of individuals who died from asthma when compared to individuals who died from other causes. Previous studies demonstrated that HK-1, which is produced from multiple sources including the bronchi, macrophages and MCs, cause chronic allergic lung inflammation in mice via NK-1R [1, 2]. This study identified MRGPRX2 as a novel GPCR for HK-1 in human MCs. Thus, MRGPRX2 may contribute to the development of asthma and may serve as a novel target for the modulation of this chronic inflammatory disease.

## Abbreviations

HK-1: Hemokinin-1
MC(s): Mast cell(s)
MRGPRX2: Mas-related G protein coupled receptor X2
NK-1R: Neurokinin-1 receptor
SP: Substance P
